# Restraint in somatic healthcare: how should it be regulated?

**DOI:** 10.1136/jme-2023-109240

**Published:** 2023-10-18

**Authors:** Amina Guenna Holmgren, Ann-Christin von Vogelsang, Anna Lindblad, Niklas Juth

**Affiliations:** 1 Department of Learning, Informatics, Management and Ethics (LIME), Karolinska Institutet, Stockholm, Sweden; 2 Heart, Vascular and Neuro Theme, Karolinska University Hospital, Stockholm, Sweden; 3 Department of Clinical Neuroscience, Karolinska Institutet, Stockholm, Sweden; 4 Department of Public Health and Caring Sciences, Uppsala University, Uppsala, Sweden

**Keywords:** Ethics, Coercion, Policy, Decision Making

## Abstract

Restraint is regularly used in somatic healthcare settings, and countries have chosen different paths to regulate restraint in somatic healthcare. One overarching problem when regulating restraint is to ensure that patients with reduced decision-making capacity receive the care they need and at the same time ensure that patients with a sufficient degree of decision-making capacity are not forced into care that they do not want. Here, arguments of justice, trust in the healthcare system, minimising harm and respecting autonomy are contrasted with different national regulations. We conclude that a regulation that incorporates an assessment of patients’ decision-making capacity and considers the patient’s best interests is preferable, in contrast to regulations based on psychiatric diagnoses or regulations where there are no legal possibilities to exercise restraint at all in somatic care.

## Introduction

 An essential ethical principle in somatic healthcare is that people have the right to decide over their own body and life.^1^ This principle of autonomy and bodily integrity is considered so fundamental that liberal democratic countries have incorporated the principle in their constitutions.^2 3^ Another important ethical principle of healthcare is to provide safe care with the aim of doing good.^1^ These principles may come into conflict when patients are at risk for harm, either when their own actions expose themselves to harm or by their choice to refuse needed care. Healthcare professionals are then faced with the dilemma of either using restraint or risking that patients will harm themselves.^4^

Compulsory care is defined as the intentional restriction of a patient’s options by physical or medical means, manipulations, or threat of punishment.^5^ Restraint is understood here as the use of chemical or physical methods to force the patient to behave in a manner inconsistent with his or her own wishes. In this paper, the term restraint will be used to encompass both physical and chemical restraint unless otherwise explicitly stated.

Restraint is used in somatic healthcare settings in many countries.^6 7^ However, studies of its prevalence show great variation.^7 8^ Although this may be explained (at least to some extent) by differences in definitions, data collection techniques and choice of empirical material, some settings are over-represented, such as units where patients with reduced decision-making capacity are being cared for.^9 10^ One such well-researched setting is intensive care,^11^ and one study conducted at 34 intensive care units in nine European countries showed that 39% of all patients had been exposed to restraint.^8^

A common notion is that restraint should be avoided, and if used, then only as a last resort in situations where patients who lack decision-making capacity risk harming themselves.^11 12^ Restraint in somatic care is mainly used when healthcare professionals perceive that there are no other options available to protect patients from harm, and is justified with the argument of being in the best interest of the patient.^11 12^ However, studies supporting the effectiveness of such restraint are lacking. On the contrary, restraint aimed at protecting patients from harm is sometimes ineffective or even counterproductive, and both physical and psychological harms have been reported.^13 14^ For instance, patients subjected to restraint may be at risk for physical injuries such as fall accidents, pressure ulcers, and side effects of sedative medications.^6 13^ Patients have described long-lasting and vivid memories of restraint in somatic care, as well as being negatively affected by their experiences.^14^ Several studies have also reported on cases of death caused by physical restraint in somatic care.^15 16^ The studies from somatic care settings are buttressed by an extensive literature reporting on physical and psychological harm caused by restraint in psychiatric care.^17 18^ These studies indicate that the instances where restraint is justified based on the best interest of the patients may be quite rare. Furthermore, the use of restraint may also be stressful for healthcare professionals, and previous research has shown that it is perceived as challenging.^11 19^

Considering the significant violation of integrity and autonomy, as well as the negative effects that restraint may have on patients and healthcare professionals, it is important to scrutinise how it is regulated in somatic healthcare. Presently, countries have chosen different paths. For instance, some have incorporated regulations concerning restraint in somatic care with regulations concerning restraint in psychiatry, while others have different laws on restraint for somatic care and psychiatry.^20^,^23^ If and how patients’ decision-making capacity matters also differs between countries. One overarching problem when regulating restraint is to ensure that patients with reduced decision-making capacity receive the care they need while at the same time ensuring that patients with a sufficient degree of decision-making capacity are not forced into care that they do not want. In this article, we argue that regulations that incorporate an assessment of patients' decision-making capacity and considers the patient’s best interest are preferable, in contrast to regulations based on psychiatric diagnoses or regulations where there are no legal possibilities to exercise restraint in somatic care at all. We will begin by describing the current regulation of restraint in some European countries. We will then continue by arguing why restraint in somatic care should be allowed to some extent, followed by a discussion on how it should be regulated.

## Current regulation of restraint

In a democratic society where human rights are taken seriously, some core principles are central, often summarised as ‘the rule of law’. Some of these principles are legality, foreseeability, equality before the law and access to justice. The implementations of these principles are different in different legal systems but are nevertheless crucial when limiting individual rights and freedoms.^24^

To elaborate and argue for regulations based on decision-making capacity and best interest, we find it important to first clarify and describe three examples of current European regulations on restraint. The described regulations are chosen because they demonstrate the difficulties that can arise in regulating the care of patients with impaired decision-making capacity who resist care, and they provide a good starting point for our arguments. The purpose of describing the three regulations is not to provide a thorough proper legal analysis but to illustrate different approaches when regulating restraint. The advantages and disadvantages of the three models will not be described in this section but will be developed later in the paper.

First, we take the example of Swedish regulations, which have proven to be problematic when it comes to providing somatic care to people with reduced decision-making capacity.^6 12 19 25^ There are currently no specific statutes that in detail regulate the permitted use of restraint of patients in somatic healthcare, except for a general rule on emergency cases in the Swedish Criminal Code.^26^ According to the Patient Act, healthcare professionals are allowed to provide healthcare without a patient’s consent in situations when a patient is unconscious and there is an acute or severe danger that threatens the patient’s life or health.^22^ This rule does not, however, imply that restraint measures are allowed in other situations. However, if a patient who permanently or temporarily has lost their decision-making capacity opposes treatment, there is no legal basis for restraint if it cannot be motivated by a severe psychiatric condition in accordance with the Swedish Compulsory Mental Act.^27^ The problems that may arise concern, for example, people with long-term impaired decision-making capacity, but which cannot be classified as a severe psychiatric condition. Here we find, for example, people with cognitive disorders or people with acquired brain injury. Hence, under current Swedish regulation there is a severe risk that patients with impaired decision-making capacity who oppose treatment will not receive the healthcare they need.

Unlike Sweden, Norway has chosen to allow restraint in somatic healthcare. The decision to use restraint is based on an assessment of the patient’s decision-making capacity and the patient’s best interests.^21^ The regulation states that before restraint may be used other measures must be tried first. If these measures do not have the desired effect, restraint may be used if a physician or a registered nurse assesses that failure to provide healthcare may lead to harm for the patient. The Norwegian regulations also state that the restraint measure should be proportionate to the patient’s need for care and that the restraint should be in the best interests of the individual patient.^21^ The restraint regulation for somatic healthcare only applies to somatic disorders. Compulsory care in psychiatric care is regulated separately.

A similar regulation to the one in Norway is the regulation in England and Wales, namely the Mental Capacity Act (MCA).^20^ One difference between the Norwegian law and the MCA is that England and Wales have no separate regulation for psychiatric and somatic healthcare, and the MCA applies to both. This means that patients with reduced decision-making capacity may be subjected to restraint regardless of whether the underlying condition is a severe mental disorder or a somatic disorder. In addition to the requirement for the assessment of decision-making capacity, there is also a requirement that the restraint should be in the patient’s best interest. The decision-making process is guided by five principles^20^:

1. A presumption of capacity—it must be assumed that all people have the capacity to decide for themselves.
2. Individuals make their own decisions—every effort should be made to encourage and support people to make decision for themselves.
3. Unwise decisions—people have the right to make decision others may find unwise.
4. Best interests—everything that is done must be done on behalf of the person’ best interest.
5. Least restrictive option- decisions or actions that interfere the least with the person’s freedom should be used.

## Should restraint in somatic healthcare be subject to regulation, but allowed to some extent?

In this paper, we presume that there are situations involving patients with reduced decision-making capacity where use of restraint may be the most defensible course of action.^5^ Accordingly, the following discussion will focus on questions regarding why, when and how restraint should be regulated and used.

We start out with the question of whether countries should implement a model with a general ban on (almost) all restraint in somatic care (like the Swedish one) or some other kind of regulation where restraint in somatic care is, to some extent, allowed and regulated (eg, it is specified when and how restraint is allowed) (like the Norwegian or English/Welsh kind). We argue that the latter is preferable.

As already mentioned, the default position in liberal democracies is that healthcare should be provided on a voluntary basis, but also that those in need of care should have access to the care needed.^28^ A general ban on restraint in somatic healthcare challenges such access because healthcare can only be provided to those who can consent, thereby discriminating against people with reduced decision-making capacity. We will elaborate on these arguments below.

### Justice-based arguments

Justice in healthcare is often discussed in terms of allocation of resources, with competing conceptions of just distribution. However, behind all conceptions there is a generally accepted fundamental core notion of justice, sometimes called the formal principle of justice, saying that equal cases should be treated equally.^29^ Or, put another way, in order to justify that two (or more) cases are treated differently there must be a relevant difference between them. It is simply this fundamental and formal sense of justice that we are invoking in this context.

In situations where healthcare professionals find it necessary to use restraint, the Swedish model on restraint results in difficulties in securing equal treatment, transparency, legality and foreseeability. These difficulties arise not only on a group level, but also when it comes to the treatment of individual patients. Let us elaborate on why that is so.

Restraint is sometimes needed to avoid serious harm for patients in somatic healthcare or, at least, healthcare professionals are convinced that this is so.^19^ Therefore, despite a ban, restraint is likely to be exercised in somatic healthcare and, again, the empirical evidence that this is indeed the case is solid.^6 19 25^ However, how, when and why restraint is used, that is, the actual exercise of restraint, is likely to be more arbitrary if there is a general ban on restraint. There are two reasons for this: first, when there are no officially accepted criteria for when something is allowed to be done, people must act according to their own values and preferences. When it comes to restraint in somatic care, this has empirical support: healthcare professionals hold and act on different notions regarding how, when and why restraint is used.^12 25^ This dependence on individual healthcare professionals’ own assessments makes the decision to use restraint arbitrary.^12^ Second, different views among healthcare professionals result in a lack of consistency and difficulties in anticipating and determining how, when and which measures are to be used.^12^

Although the case of England and Wales indicates that the arbitrariness of the decision-making process regarding restraint cannot be eradicated,^30^ regulations on restraint may support the development of common policies and guidelines with the potential to secure legal certainty and equal treatment in the decision-making process.

### Avoiding patient harm

One might argue that saving lives and alleviating suffering are the main obligations of the healthcare system and that healthcare legislation should support healthcare professionals in fulfilling these obligations.^31 32^ This resonates with the ethical views of healthcare professionals: when scrutinising their reasons for using restraint, the main reason reported is that it is used to protect patients from harm.^12 30^ Hence, regulations on restraint in somatic healthcare based on avoidance of harm, which is what we are proposing, is likely to be looked favourably on and be abided by healthcare professionals. On the other hand, without legal possibilities to exercise restraint, healthcare professionals are forced to make decisions about whether to follow the law (and not use restraint) or risk severe patient injury, including even death.^13 15^ The sorts of harm might differ between healthcare contexts, but the most common reasons in intensive care and geriatric care are to stop patients from pulling at invasive devices, to prevent patients from falling or to prevent them from leaving the ward.^11 33^

Hence, there is no guarantee that regulation permitting some restraint will result in a more positive risk–benefit ratio than a regulation banning all restraint. We propose that this could only be decided by looking into the details of the regulations in question as well as conducting further empirical studies on their effects. We explore the hazards of allowing some restraint further below (see the Alternatives to restraint section).

Another issue that has arisen in recent studies is that restraint is sometimes used as a convenient option for healthcare professionals rather than for the benefit of the individual patient.^12 34^ Regulating restraint in somatic healthcare may discourage this sort of justification and use. The violation of integrity and autonomy, and the possible negative side effects, require that restraint is used properly and for the right reason. Regulations may stipulate conditions promoting such use and may incorporate a demand for education and evaluation, thereby ensuring patient safety.

### Lack of respect for patients’ rights

An important aspect concerning transparency and legal security is the possibility to over-rule decisions on restraint. For example, in Norway, England and Wales, patients have the right to appeal decisions on restraint in somatic healthcare.^20 21^ If patients or relatives are dissatisfied with decisions on restraint, they have the right to demand a second opinion by an objective party, and then has the right to over-rule the decision. In contrast, when restraint is used without legal support, the possibility of appealing a decision to use restraint becomes more difficult.

According to recent studies from Sweden, restraint use is often hidden, sometimes deliberately. Patients exposed to restraint are not informed after the events,^19^ and documentation in medical records is lacking; for example, fewer than 11% of all restraints at a neurosurgical department were documented in the patients’ electronic medical records.^6^

However, lack of documentation of restraint has also been reported from countries with a more permissive regulation on restraint.^35 36^ Furthermore, it has also been reported that healthcare professionals fail to report adverse events associated with restraint use.^35^

In Sweden, healthcare professionals have expressed feelings of guilt and an awareness that restraint may be questionable from a legal perspective and have reported that they, therefore, refrained from writing about it in the patient’s medical records.^19^ As a consequence, the applied restraints are concealed from the exposed patients. This is problematic since the process of carrying out open restraint measures typically leaves room for patients to express their views, to invoke applicable rights, and to potentially appeal the measures. As mentioned earlier, the Swedish model leads to an absence of transparency, legality and foreseeability. This is, again, problematic from the point of view of justice. But this may also create insecurity regarding the right to autonomy for patients and their right to have their voice heard.

A general unspecified ban on restraint in somatic healthcare might have a negative impact on trust in the healthcare system, at least among some patients who could be subject to decisions about restraint (by trust we mean the trustor’s confidence that healthcare professionals are competent and acting in the best interests of the trustor). Due to the negative impact of restraint on integrity and autonomy, a transparent and legally predictable decision-making process is required; it needs to be clear as to what grounds decisions on restraint are made. Without regulation that allows and regulates restraint, there is a significant risk that patients who have decision-making capacity will be forced into care that they do not want, which may negatively affect their trust. Furthermore, there is also a significant risk that patients with reduced decision-making capacity will not receive the care they need. Both these groups of patients might be less inclined to trust in healthcare. At least, with a general ban on restraint, they have no reason to trust that healthcare will act accordingly.

### Alternatives to restraint

As previously mentioned, there are potential harms with removing a general ban on restraint. To understand one such hazard, it is vital to consider what the purpose of not allowing restraint in somatic healthcare might be. In Sweden, the general ban on restraint in somatic healthcare aims to encourage alternative solutions to restraint.^37^ The main hypothesis is that a ban on restraint in somatic healthcare creates an inducement to avoid restraint and encourages healthcare professionals to create alternative ways of caring for patients with challenging behaviours. Hence, allowing some restraint may reduce the incentive to look for alternatives to restraint.

While we acknowledge that this is a potential upside with a general ban on restraint in somatic healthcare, there may be regulatory means to counter this problem. In fact, a demand to investigate alternatives may be part of a regulation, and in countries with more permissive legislation there are requirements that other measures must be tried before using restraint.^20 21^

Another important difference between countries where restraint is permitted under certain conditions and countries where it is prohibited is that in countries where it is permitted healthcare professionals are allowed and encouraged to undergo training in how and when restraint is to be used. In countries where restraint is prohibited, such training is also prohibited, which forces healthcare professionals to create their own rules and methods for when and how restraint is to be used.

### Minimising unnecessary restraint

One might argue that a ban on restraint may decrease the likelihood of restraint being used, and that if it is used it will be to a lesser degree and less restrictive. Although there is currently no solid evidence suggesting that this is the case, this may be so. However, the risk of an extensive use of restraint could instead be handled through a thorough regulation and a requirement to report all restraint used.

Healthcare professionals often argue that they only use restraint as a last resort, but as already mentioned it is sometimes also used as a convenient option.^12^ It has been reported that caring for patients with challenging behaviour is draining and difficult and that restraint is a method to secure a peaceful working environment.^12 34^ A regulation clearly stating the conditions for restraint use, that is, that restraint should only be used for the benefit of the individual patient, together with a requirement to record and report all restraint measures to healthcare authorities, could discourage this aspect of restraint use.

## How should restraint in somatic healthcare be regulated?

In the previous section, arguments for a regulation on restraint in somatic healthcare have been presented, to some extent specifying under what conditions restraint should be allowed: when the patient is not decision competent and restraint is in the interest of the patient, that is, doing more good than harm for the patient. We will now continue by discussing why regulation on restraint should be based on an assessment of decision-making capacity as well as the patient’s best interests. This kind of regulation would apply to somatic as well as psychiatric conditions. We argue that this is preferable to a regulation with different standards for different groups (ie, patients with somatic vs psychiatric conditions). Thus, we argue that in this regard the English/Welsh model is preferable to the Norwegian.

### Avoiding discrimination (of psychiatric patients)

The first question to consider is whether there are any relevant differences between mental and somatic disorders that could justify separate regulations. As described above, persons should be treated equally if there is no relevant difference between them.^29^ In the matter of decision-making capacity, no such difference appears to exist, and both somatic and mental disorders may result in reduced decision-making capacity, thereby affecting patients’ ability to decide about their own health. Impaired decision-making capacity is a symptom and not a diagnosis. According to a systematic review, there are no significant differences between psychiatric and somatic care settings in terms of proportion of incapacity.^38^ The review found that 34% of patients cared for at medical and surgical units had impaired decision-making capacity.^38^ Furthermore, physicians often underestimate the number of patients who have impaired decision-making capacity in somatic healthcare.^39^

Regulations like the one in Sweden discriminate against patients with mental disorders, who may be subjected to restraint even though they are competent to make decisions, and against patients with somatic disorders who cannot be subjected to restraint even in cases with reduced decision-capacity. There seems to be an underlying view that mental disorders are different, in some sense even exceptional, and that this justifies a different approach. However, this view echoes of old prejudices.^28^ A regulation that incorporates both psychiatric care and somatic care avoids discriminating against people based on the disorder they suffer from. Such a regulation could take either of two forms: restraint being regulated by one regulation covering all medical conditions, and restraint being legal under substantively the same criteria regardless of whether the condition is somatic or psychiatric, even though the regulations are technically separated. We will not take a stand on which of these are preferable in this context.

### Respecting autonomy (only restraining those with insufficient decision-making capacity)

We agree that regulation on restraint in healthcare should accept the principle of autonomy^40^; individuals that are considered autonomous enough to decide to refrain from care offered should be allowed to do so: soft paternalism (restraining only those who are unable to decide for themselves) is much more justifiable than hard paternalism.^5^ If so, this should be reflected in regulation and then regulation needs to differentiate between those who should be deemed as having the capacity to decide and those who do not. Accordingly, there is a need for regulation to involve assessment of decision-making capacity and a threshold of decision-making capacity.

As described above, some countries have requirements for the assessment of decision-making capacity when deciding on restraint measures.^20 21^ In England and Wales, the assessment is based on well-known and accepted definitions of decision-making capacity and is carried out by licensed healthcare professionals.^20^ In short, the patient must be able to understand relevant information, appreciate the situation and understand its consequences, comprehend information rationally and communicate their choice.^41^ Here, patients capable of making decisions about their own health are allowed to do so, thus protecting patients’ autonomy. A structured assessment of decision-making capacity may also discourage arbitrary assessments coloured by healthcare professional’s own preferences or values, at least to some extent. Furthermore, it promotes transparency and legal certainty when it is clear when and how decisions about restraint are made.

However, the assessment of decision-making capacity may not always be a matter of black and white, which has been voiced by critics in England and Wales.^42^ For instance, what level of decision-making capacity people should have in order to make decisions about their own health is difficult to assess.^42 43^ In addition, these difficulties are partly related to the fact that many patients have decision-making capacity regarding certain decisions but not others. Therefore, assessing decision-making capacity needs to be decision-specific. Furthermore, decision-making based on personal values and difficulty in avoiding personal bias has also been reported.^44 45^ Dealing with these difficulties can be remedied by better training, at least to some extent, and the use of specific instruments.^46^ Assessing mental capacity requires both clinical and ethical knowledge and skills.^44^

Despite struggles to assess decision-making capacity, healthcare professionals still value the principles underlying the MCA and have reported a positive impact on practice, where the MCA promotes person-centredness, equality and inclusivity.^43^ Also, the number of grey-zone cases where assessment of decision-making capacity is difficult should not be exaggerated: at least in some areas of somatic care, such as neurosurgery, those deemed lacking decision-making capacity are clear cases.^47 48^

Although we will not argue this point as thoroughly as deserved in this context, it is important to distinguish between the capacity to decide and the risk of harm that a decision may bring. We would, therefore, reject a principle of autonomy stating that a higher level of decision-making capacity is needed for decisions involving greater risks for the patient (although it may be more important to establish that the patient has sufficient decision-making capacity if much is at stake).^5^ On the contrary, it may be argued that the more that is at stake for the patient, the more important it is that the patient gets to decide.

In summary, we believe that the introduction of regulations based on decision-making capacity requires a long-term and elaborate implementation of assessment features. In difficult cases, there should be a support system for healthcare professionals, as well as a possibility for patients and relatives to appeal against the assessments that are made.

### Minimising harm (only restraining when it is a benefit for the patient)

That healthcare should be provided with the aim of promoting the best interests of patients is an established and accepted principle in healthcare. The care provided must aim to promote the patient’s health and/or alleviate suffering. When caring for a patient who has decision-making capacity, the patient’s own wishes and preferences govern what is the best option for the patient. This means that the patient may refuse treatment that is considered important for their health and well-being. In contrast, patients who lack decision-making capacity require others to make decisions for them. How, by whom and on what grounds these decisions are to be made is a major source of debate.^49^

An important aspect in the decision-making process is to identify what it is that should be protected. In other words: restraint needs to be proportionate to what is at stake. For instance, more restraint can be used against a patient who is trying to jump out of a window compared with a patient trying to pull out a peripheral venous catheter, which may involve discomfort but not a fatal outcome. Recently published studies have shown that healthcare professionals who use restraint describe that they make an assessment of which options provide the best outcome for the patient, but that the assessment depends on the person who makes it.^12^ Therefore, regulations should include and clarify this aspect in order to increase legal certainty and ensure safer care for patients with reduced decision-making capacity.

An important argument for considering the best interests of individual patients when deciding about restraint is that this prevents restraint from being used because resources are scarce. Recently published studies have shown that restraint is often used when healthcare professionals need to prioritise between patients and when nurses have many patients.^12 34^ A restless patient can influence how much time the healthcare professionals can spend on other patients, and by using restraint the restless patient can be controlled, which gives the healthcare professionals more time for other patients.^12^ If restraint were only allowed to be used for the sake of the individual patient, restraint would in most of these cases not be used. Regulations in which there is a requirement to only use restraint when it is in the individual’s best interests ensures that patients with a high need for care receive the care they need.

As we have seen, there are several arguments in favour of including the patient’s best interest as a part of a regulation on restraint in healthcare. However, there are situations where such a criterion could be too narrow. For instance, there may be situations where restraint for the sake of other people could be justified, such as a patient acting violently or threatening the safety of other patients and/or healthcare professionals. Because this is an important topic that deserves a thorough investigation, it will not be addressed further in this paper.

Another issue that should be addressed further is to what extent restraint measures are more justified the less harmful (or wrongful) the restraint is. For instance, diverting someone’s attention in order to administer a needle without consent may be thought less morally problematic than tying them down for the same purpose. Perhaps this should be reflected in regulation as well. We also leave this issue for further inquiry.

## Conclusion

Restraint is common in somatic healthcare settings, but regulations of its use differ between countries. In this paper, arguments of justice, patients’ rights, minimising harm and respecting autonomy are contrasted to different national regulations. We conclude that regulations incorporating an assessment of the patient’s decision-making capacity and considering the patient’s best interest is preferable, in contrast to regulations based on psychiatric diagnoses or regulations excluding the use of restraint in somatic healthcare.

## Data availability statement

No data are available.
